# Morphological and physicochemical diversity of snow algae from Alaska

**DOI:** 10.1038/s41598-020-76215-x

**Published:** 2020-11-05

**Authors:** Marta J. Fiołka, Nozomu Takeuchi, Weronika Sofińska-Chmiel, Sylwia Mieszawska, Izabela Treska

**Affiliations:** 1 Department of Immunobiology, Institute of Biology Sciences, Maria Curie-Skłodowska University, Akademicka 19, 20-033 Lublin, Poland; 2 Department of Earth Sciences, Graduate School of Science, Chiba University, Chiba, Japan; 3 Analytical Laboratory, Institute of Chemical Sciences, Maria Curie-Skłodowska University, Lublin, Poland

**Keywords:** Cell biology, Chemical biology, Microbiology, Environmental sciences, Chemistry

## Abstract

Snow algae are photosynthetic microbes growing in thawing snow. They usually show various morphological cell types. The aim of this study was to carry out microscopic and spectroscopic analysis of different forms of cells of snow algae collected on glaciers in Alaska. Four different shapes of algal cells were observed with the use of bright field LM (Light Microscopy), DIC (Differential Interference Contrast), EDF (Extended Depth Focus), fluorescence microscopy, and SEM (Scanning Electron Microscopy). The cells exhibited the strongest autofluorescence after the exposure to 365-nm excitation light, and the intensity differed among the cell types. Zygotes (cysts) showed the most intense fluorescence. Acridine orange staining revealed the acid nature of the algal cells. The use of Congo red and Calcofluor white fluorochromes indicated differences in the structure of polysaccharides in the cell wall in the individual types of algal cells. FTIR (Fourier-Transform Infrared Spectroscopy) analyses showed the presence of polysaccharides not only in the algal cells but also in the fixative solution. The presence of polysaccharides in the extracellular algal fraction was confirmed by X-ray dispersion spectroscopy (EDS), X-ray photoelectron spectroscopy (XPS), and scanning electron microscopy imaging (SEM). The differences observed in the structure of the cell wall of the different forms of red snow algae prompt further analysis of this structure.

## Introduction

The red or pink color of snow caused by blooms of snow algae is commonly observed during summer in alpine and coastal polar regions. It is also called watermelon snow, as it not only has the color of watermelon flesh but also emits a delicate watermelon aroma when crushed^1^. Snow algae have long been the subject of interest and intensive research conducted by many scientists on all continents^2^. The red color of algae is associated with the carotenoid astaxanthin found in algal cells outside chloroplasts. This secondary pigment probably protects photosynthetic pigment-protein complexes against damage by strong solar radiation^3–6^. Snow algae can cause reduction of snow surface albedo. Albedo is a ratio of the reflected radiation to the total incoming solar radiation at the snow surface; thus, albedo reduction induces higher snow melting rates. The abundance of red-pigmented algae may be associated with solar insolation, as shown by the dominance of red-pigmented algal cells in strongly insolated sites. It is assumed that the intensity of red pigmentation in algal cells is also affected by the availability of nutrients^4,7^.

Red snow algae live in a layer of liquid water covering snow grains or between grains. The concentration of cells in colored snow fluctuates and the intense red color indicates their high concentration^8,9^. The conditions of the habitat where snow algae grow are extreme in terms of temperature. These microorganisms have a unique ability to live actively at around 0 °C even with very low nutrients^6,10,11^. Some forms of development have thickened cell walls and can have an additional outer mucous coating. Thanks to these adaptations, these forms can survive dry and warm periods, i.e. after snowmelt^6^.

Algae involved in the phenomenon of red snow represent various species; however, most often, *Chlamydomonas nivalis* is responsible for the red snow. This snow alga was described as dominant in the algal community colonizing Gulkana Glacier and Harding Icefield in Alaska^13,14^. The red snow appearing in the snow area along the snow line on the glaciers can be revealed by analysis of satellite images^13^. *Chlamydomonas nivalis* has recently been described as a member of a new genus *Sanguina* with two species: *S. nivaloides* and *S. aurantia*^15^. Cysts of different species of snow algae can occur together in the same area and can spread locally in the same polar regions each year^2^. The difficulties in identifying snow algae make the taxonomy of these microorganisms a subject of intense molecular research^16^.

*Sanguina* spp. (formerly *Chlamydomonas nivalis*) occurs in various areas reaching the border of snow that never completely melts. Interpretations of experiments on field-collected cells are limited due to their short-term nature, and environmental conditions occurring in nature, where there is a high variability of abiotic conditions, are hard to simulate. Nevertheless, such attempts should be made to explore these species. *Sanguina* spp. spend most of their life cycle as cyst cells, which are more resistant to harsh conditions. These forms do not divide on the snow surface throughout the summer season but show metabolic processes. Transformation into cyst cells goes along with the accumulation of storage metabolites such as pigments, lipids, and sugars^17,18^. As shown by studies of snow algal blooming, algal cells are often found in this form^19^.

The analyzed microalgae have become interesting from the biotechnological point of view, as they are a source of thermostable enzymes, and have gained commercial interest as a source of astaxanthin^20^. Snow algae may be a potential source of pharmaceuticals, dietary supplements, or cosmetic products^21^. Cultivation of microalgae creates opportunities for the production of food and fuel commodities, but the low growth rate of many species can hinder this process^22^. In the era of global warming and the rapid melting of snow and ice, snow algae are of particular interest to scientists, as they contribute to environmental changes on Earth. Snow algae are an interesting research object for ecologists, climate scientists, microbiologists, and chemists. They are morphologically interesting, diverse in forms, not fully understood, and still very intriguing.

The aim of our research was to carry out morphological and physicochemical analysis of various morphological types of red snow algal cells from selected sites in Alaska using microscopic and spectroscopic techniques. The different types of algal life cycle were visualized using various light and scanning electron microscopy techniques and compared. The presence of intracellular and extracellular polysaccharides in the algae was examined by spectroscopic analyses as well.

## Materials and methods

### Samples

**Figure 1:**
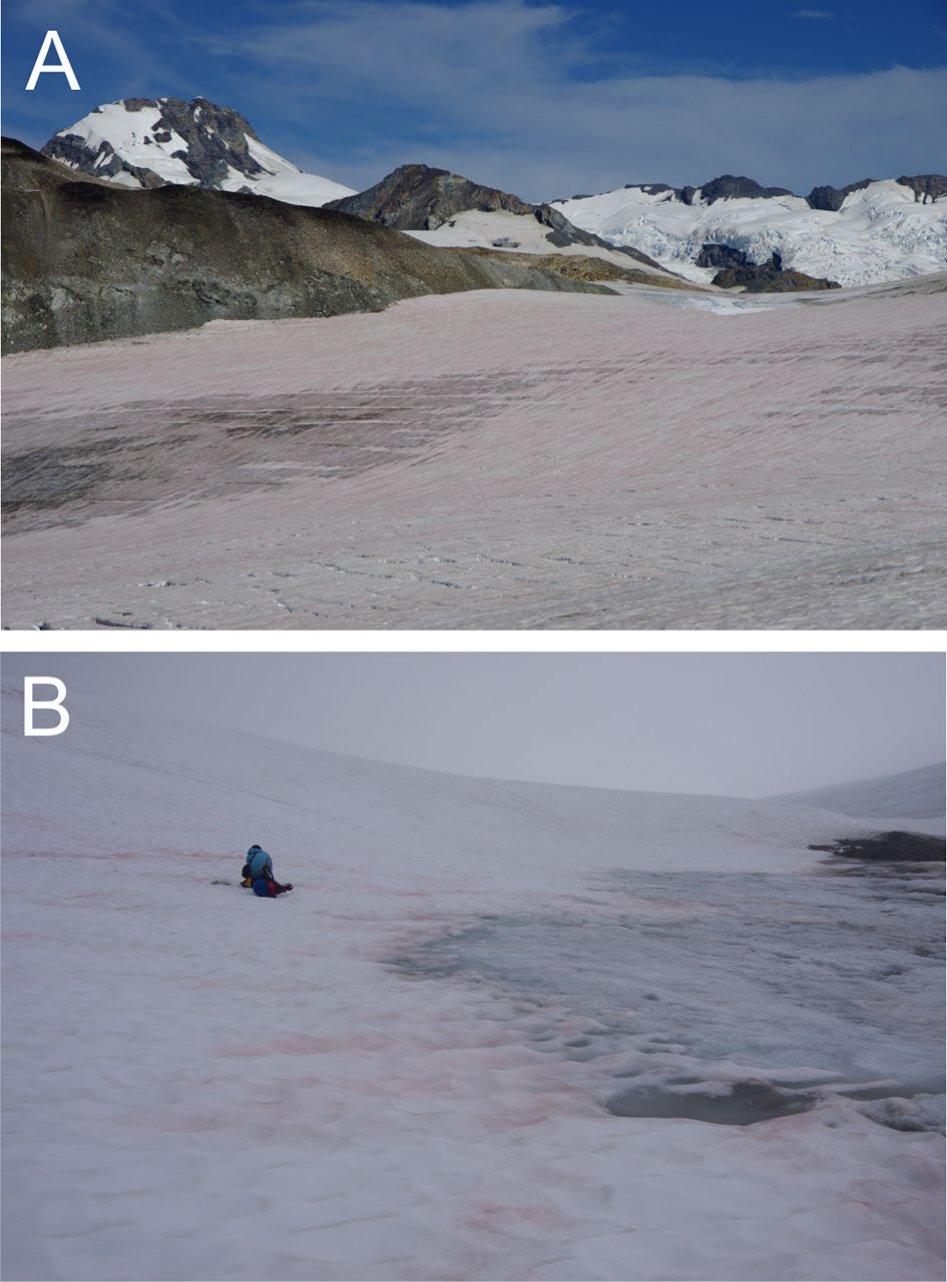
Red snow algae on Gulkana Glacier (**A**) and on Harding Icefield (**B**) in Alaska. The photos were taken on: (**A**) August 5, 2015; (**B**) August 9, 2015.

Red snow samples from two glaciers in Alaska (USA) were analyzed in this study. The samples were collected with a stainless-steel scoop (1–2 cm in depth), melted, and preserved in a 3% formalin solution (Fujifilm Wako Chemicals) in 30-ml polyethylene bottles. The study sites were Gulkana Glacier [position: N63.28697, E145.39575, elevation, 1796 (m)] and Harding Icefield [position: N60.17649 E149.73234, elevation, 1073 (m)]. Samples were obtained from August 5^th^ to 9^th^, 2015. Images of the red snow from Gulkana Glacier and Harding Icefield are shown in Fig. 1. The pH of the collected snow was 6.3–6.4. The algal cell concentration in the snow was 3.2 ± 1.9 × 10^4^ cells mL^−1^ (Gulkana Glacier) and 5.2 ± 2.6 × 10^4^ cells mL^−1^ (Harding Icefield).

### Light microscopy

Bright field light microscopy, DIC microscopy (Differential Interference Contrast), and EDF microscopy (Extended Depth of Focus) were used for imaging the algal cells. 2 µl of the cell suspension were applied to the microscope slide. The cells were observed under a Zeiss Axiovert 40CFL light microscope Carl Zeiss (Germany). EDF microscopy photos were taken with an MA200 Nikon (Japan) optical microscope with inverted optics equipped with a confocal attachment that works with three lasers. 400–600 cells of each form of algal cells were measured using the ImageJ application (National Institute of Health, USA).

### Profilometric analysis of algal cells

Optical profilometry is a method for precise spatial imaging of the surface microgeometry of selected materials. The surface texture, especially its roughness and waviness, is characterized by many parameters, the most important of which are the amplitude-height parameters. The optical profilometer facilitates quick and contactless reproduction of surfaces in three dimensions at a low magnification. The profilometric analysis of algal cells preserved in a 3% formalin solution was carried out using a Contour GT optical profilometer from Bruker (Germany). The tests were performed in VSI (Vertical Scanning Interferometry) mode. The analyses were carried out with a validated method. Three repeats of measurements were conducted for each cell type. The roughness parameters of the four types of algal cells are presented in Table 4.

### SEM of algal cells

Scanning electron microscopy (SEM) was used to visualize the morphology of the algal cells more accurately. Cells preserved in a 3% formalin solution were washed in 0.1 M phosphate buffer pH 7.0, fixed with 4% glutaraldehyde, washed again in phosphate buffer, incubated in a 1% OsO_4_ solution for 1 h, and dehydrated with an acetone gradient (successively at 15%, 30%, 50%, 70%, and 100% for 20 min). The preparations were then dried using silica gel prepared in the dryer (for 2 h at 121 °C) and coated with gold using a K550X sputter coater from Quorum Technologies (UK)^23^. The samples were analyzed using a Tescan Vega 3 scanning electron microscope from Tescan Analytics (Czech Republic). The images were selected after three independent analyses were performed.

### Autofluorescence analysis of algal cells

The algal cells were observed using light microscopy upon excitation at three different wavelengths with 365 nm (Filter Set 02), 470 nm (Filter Set 13), and 546 nm (Filter Set 15) filters from Carl Zeiss (Germany). A Zeiss Axiovert 40CFL light microscope from the same company was used to observe and photograph the cells. Four cell forms were imaged, and cells with different degrees of illumination were compared with each other. The cells were photographed at 40 × magnification. Fluorescence was measured using the ImageJ program. To measure the fluorescence of the algal cells, the image was converted to an 8-bit grayscale microscopic image to obtain five-digit fluorescence values. Relative Fluorescence Unit (RFU) fluorescence is the sum of the intensity and luminosity of all pixels measured in the microscopic image. The analyses involved 288 cells.

### Analysis of algal cells after application of fluorochromes

The algal cells in a 3% formalin solution were stained with three types of fluorochrome: Congo red, Acridine orange, and Calcofluor white (Table 1). Pictures were selected after analysis of 100 cells from each sample. Congo red (Sigma) is a water-soluble organic compound yielding a red colloidal solution. It is usually used to stain β-glucans in the fungal cell wall^24^; however, this dye also binds to the polysaccharides of the algal cell wall and has an especially strong affinity for cellulose fibers^25^. The algal cell suspension in 3% formalin was incubated with a 1% Congo red solution (in a 1:1 volume ratio) for 10 min at room temperature in complete darkness. Then, 2 μl of the mixture were applied to the microscope slide. Unstained cells were imaged at a magnification of 40 ×. The cells were imaged with a Zeiss Axiovert 40CFL light microscope from Carl Zeiss (Germany).

**Table 1:** Fluorochromes used to dye algal cells.

Dye reagent	Excitation wavelength (nm)	Color of fluorescence	Visualized structures
Congo red	543	Red	Cell wall polysaccharides
Acridine orange	470	Green (cytoplasm), orange (acidic compartments)	Cell compartments with acidic pH
Calcofluor white	456	Blue	Cell wall cellulose

Acridine orange (Sigma) is a common fluorochrome used to stain living and fixed cells. At low pH, when excited by blue light, Acridine orange can differentially stain cells green. This dye is permeable to cells and interacts with DNA and RNA by intercalation or electrostatic attraction, respectively. When bound to DNA, it is very similar in spectra to fluorescein, with a maximum excitation at 502 nm and a maximum emission at 525 nm (green)^26^. All cell images were taken using immersion. Acridine orange can be used to stain acidic organelles such as lysosomes, autosomes, or vacuoles. It emits orange fluorescence inside these organelles at low pH. The red algal cell suspension was mixed with a 0.001% aqueous Acridine orange solution in a volume ratio of 1:1 and incubated in the dark for 20 min. Afterwards, the cells were imaged at a wavelength of 450 nm^26^ using the microscope mentioned above.

The Calcofluor white fluorochrome is mainly used to stain fungal cell walls, since it binds to chitin^23,27,28^. It is also known to bind to cellulose^29^. Calcofluor white (Fluka) was added to the aqueous suspension of algal cells in a 1:2 ratio. The algal cells were incubated for 10 min in the dark and imaged as specified above. It is known that algae differ in the structure of the cell wall^15^. In our experiments, this dye was used to check whether it binds to the cell wall of any of the algal forms analyzed.

### FTIR (Fourier-transform infrared spectroscopy) spectroscopy

FTIR-ATR (Attenuated Total Reflectance) spectroscopy was employed to detect and identify polysaccharides contained in algal cells. The FTIR analyses were performed using a FTIR Nicolet 8700 spectrometer from Thermo Scientific (UK) and the ATR technique with a diamond crystal in the range of 4000–400 cm^−1^ wavenumbers and 4 cm^−1^ spectral resolution. The spectra were recorded from material fixed in a 3% aqueous solution of formaldehyde, applying the solution at room temperature directly on the diamond crystal of the ATR attachment. The obtained spectra were normalized. The experiment was repeated three times.

### SEM/energy dispersive X-ray spectroscopy (EDS) analysis of polysaccharides

500 µl of the supernatant of the algal suspension in a 3% formalin solution were frozen at − 20 °C and lyophilized. The formalin solution was removed during this process. The white powder obtained after freeze-drying was imaged using SEM and its elemental composition was analyzed. Polysaccharides were imaged using a Quanta 3D FEG high-resolution scanning electron–ion electron microscope from FEI Company (USA).

The specimen was prepared by fixing the sample to the Al microscope table and transferred into the sample chamber of the microscope. The thickness of the sample was chosen so as not to obtain the Al signal from the table. The microscope system was equipped with an EDS spectrometer from EDAX (GB), and EDS measurements were conducted at 30 kV beam energy. Various locations of the sample were point analyzed and 4-point characteristic spectra were collected for each sample. The elemental composition (atomic concentration) of the investigated points was calculated with EDS.

### X-ray photoelectron spectroscopy (XPS)

X-ray photoelectron spectroscopy (XPS) measures the distribution of the energy of electrons emitted from a sample that has absorbed ultraviolet photons (ultraviolet photoelectron spectroscopy, UPS) or X-ray radiation (X-ray Photoelectron Spectroscopy, XPS)^30^. An Ultra High Vacuum multi-chamber analytical system from Prevac (Poland) was used to investigate the prevailing chemical structures in extracellular fraction the snow algae. Samples fixed on a molybdenum carrier were degassed at room temperature to high constant vacuum of ca. ~ 5 × 10^–8^ mbar in the UHV sluice system. After transferring the samples into the analytical chamber of the system, the analysis was performed by means of XPS spectroscopy. AlKα monochromatic radiation was used as a source of photoelectrons. Photoelectrons were stimulated with X-ray of the characteristic line AlKα at 1486.7 eV energy generated by a VG Scienta SAX 100 lamp with an aluminum anode with a VG Scienta XM 780 monochromator. The pressure in the chamber during the measurements was 2 × 10^–8^ mbar. The X-ray tube operating parameters were as follows: U = 12 kV, Ie = 30 mA. Photoelectrons were recorded by the hemispherical analyzer Scienta R4000. The measurements were made at the following basic parameters: operating mode-sweeping, pass energy − 200 eV, measured range of the binding energy of photoelectrons 0–1200 eV, measuring step 0.5 eV, collection time in a single step 0.2 s, and number of iterations 5. The parameters of the analyzer for the high-resolution spectra were as follows: operating mode–sweeping, pass energy − 50 eV, measuring step − 0.1 eV, and collection time in a single step − 0.667 s^31^.

500 µl of a supernatant of the algal suspension in a 3% formalin solution were frozen at − 20 °C and lyophilized. The formalin solution was removed during this process. The white powder obtained after freeze-drying was examined by XPS spectroscopy. The study aimed to identify chemical bonds present in the preparation. The experiment was repeated three times.

### Statistical analysis

Statistical analysis was performed using the Statistica application and the ANOVA Kruskal–Wallis test for nonparametric analysis. Kruskal–Wallis, H (3, N = 288) = 111.8398; p = 0.000. Statistical significance between individual cell types was analyzed.

## Results

### Light microscopy

**Figure 2:**
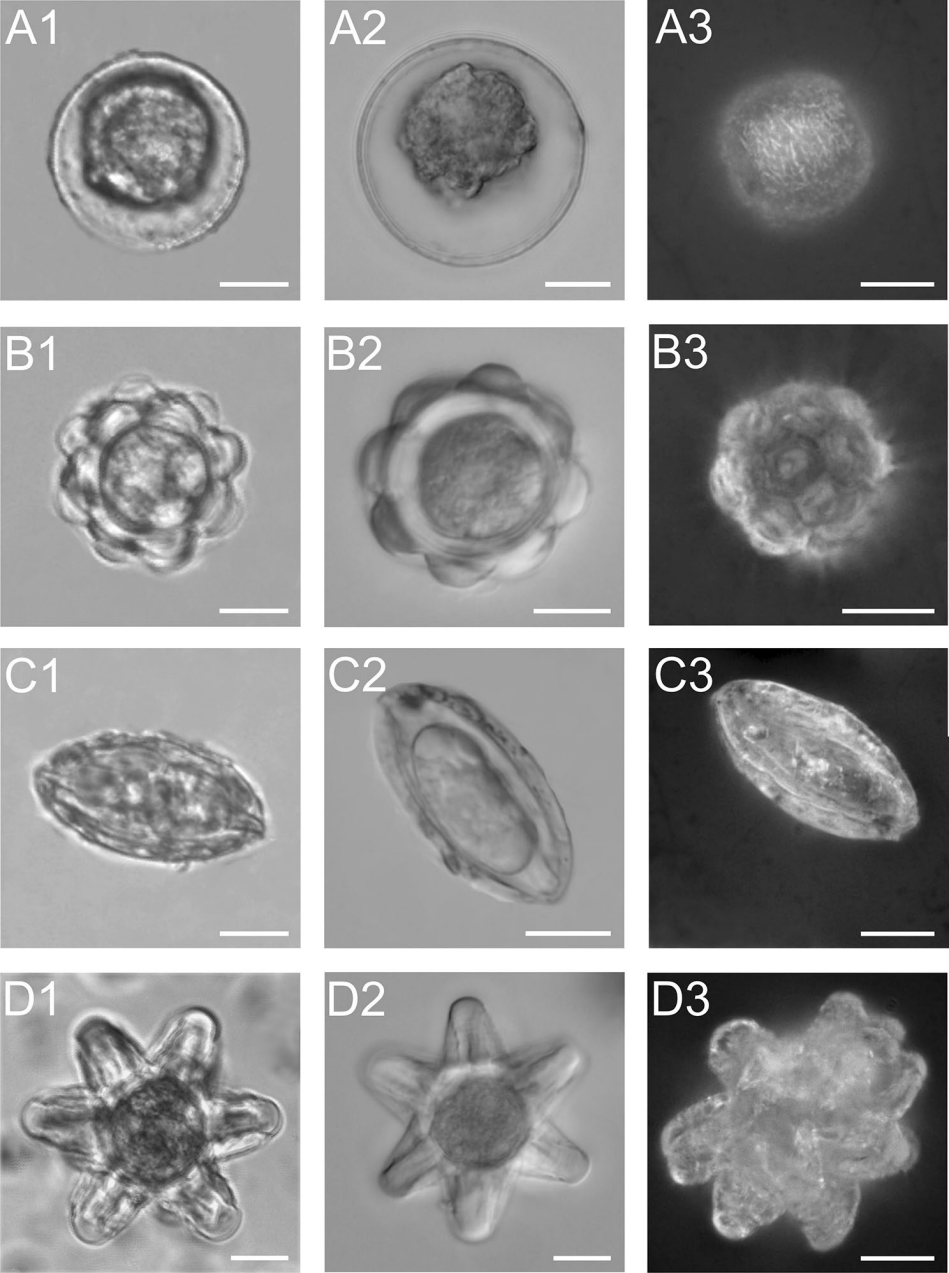
Different morphological forms of snow algae fixed in formalin (putative *Chlamydomonas* sp.^31^ or *Sanguina* sp.^15^, formerly *Chlamydomonas nivalis*) observed in Alaska. A—round cysts-spore cell, B—rosette form-hypnozygote, C—ellipsoid form-*Chloromonas* zygote, D—star form. The scale bar corresponds to 10 µm.

Preserved algal cells were analyzed using three different light microscopy techniques: transmitted light, DIC, and EDF. Four different forms or stages of snow algae were observed: Type 1: round (cysts), Type 2: rosette-shaped (cysts, hypnozygote), Type 3: fusiform (cysts of *Chloromonas* spp.), and Type 4: star-shaped (cysts of *Chloromonas* spp.) (Fig. 2 and Table 2). Type 1 cells have a clearly visible surrounding transparent coating. Moreover, as shown by the EDF analysis, the cell surface is not smooth but rough. Type 2 consists of many symmetrically arranged small rosettes, which form a flower-like shape. This effect is also visible when EDF lasers are used. Type 3 has an elongated fusiform shape visible in images C2 and C3 in Fig. 2. Type 4 has 12 spatially arranged arms. Six arms were clearly visible in DIC and bright field LM. The EDF image showed additional six arms in this form (Fig. 2).

**Table 2:** Types, shapes, and stage of the cycle of algal cells.

Algal type	Shape	Stage of cycle	Suggested species	References
Type 1	Round	Cyst	*Sanguina nivaloides/Sanguina aurantia (*formerly *C. nivalis)*	Procházková et al.^15^
Type 2	Rosette	Hypnozygote	*Chloromonas nivalis*	Müller et al.^6^
Type 3	Fusiform	Zygote	*Chloromonas nivalis*	Muramoto et al.^32^
Type 4	Star	Unknown	*Chloromonas* spp*.*	Segawa et al.^34^

### Abundance and cell sizes

**Figure 3:**
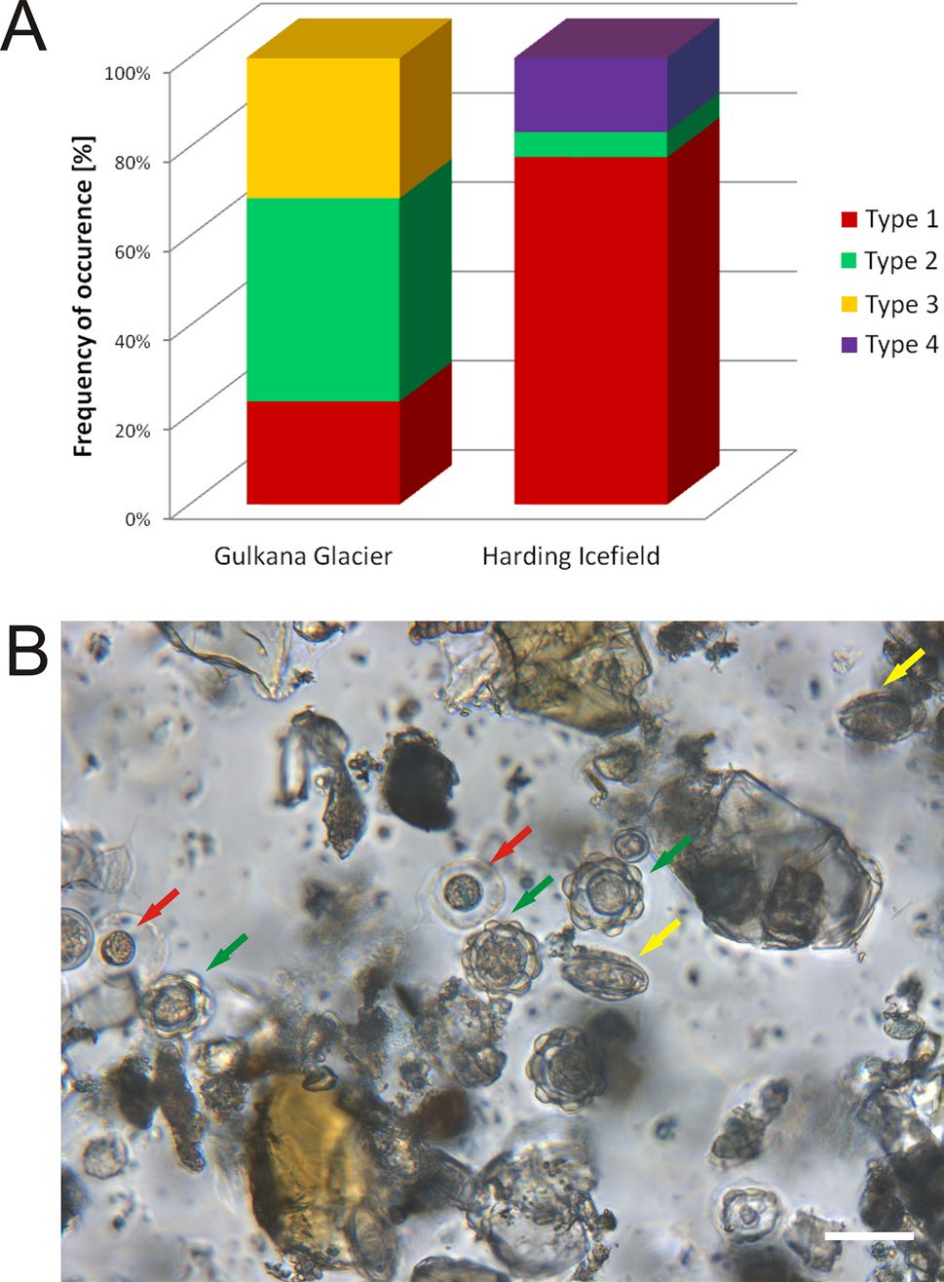
Abundance of individual forms in the analyzed samples: *S1* Gulkana Glacier (Alaska), *S2* Harding Icefield (Alaska). The image shows snow algae from Gulkana Glacier (formalin fixed). The scale bar corresponds to 20 µm.

Types 1, 2, and 3 were found in the samples from Gulkana Glacier and Types 1, 2, and 4 were found in the samples from Harding Icefield (Fig. 3). Type 2 prevailed on Gulkana Glacier (46%), and Type 1 was the predominant form on Harding Icefield, as it constituted almost 80% of all cells. Type 3 occurred on the former glacier and represented 32% of cells. In turn, Type 4 was only present on the latter glacier and accounted for 17% of all algal cells (Fig. 3).

**Figure 4:**
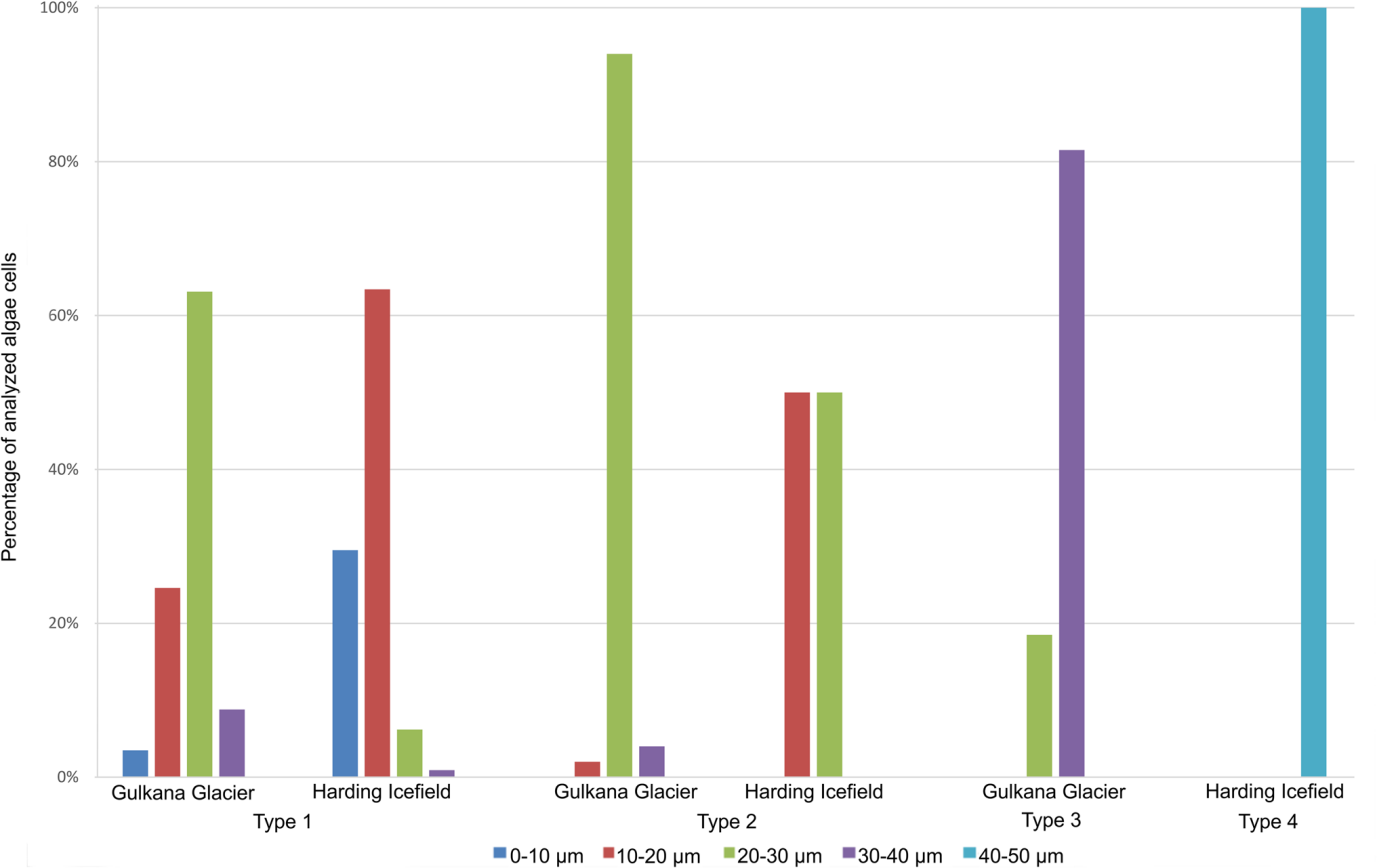
Percentage of algal cells of different sizes in Gulkana Glacier and Harding Icefield samples.

The sample from Gulkana Glacier was dominated by cells with a 20–30 µm diameter belonging to both Type 1 (63%) and Type 2 (94%). Type 3 was present in this location as well and as much as 82% of these algae had a size in the range of 30–40 µm. In the sample from Harding Icefield, Type 1 dominated in the size of 10–20 µm, Type 2 was represented in half by 10–20 and 20–30 µm cells. Type 4 algal cells were the largest and all were 40–50 µm in size (Fig. 4, Table 3).

**Table 3:** Percentage of algal cells of different size.

Cell type	Glacier	Cell size (µm)				
		0–10 (%)	10–20 (%)	20–30 (%)	30–40 (%)	40–50 (%)
Type 1	Gulkana Glacier	3.5	24.6	63.1	8.8	–
	Harding Icefield	29.5	63.4	6.2	0.9	–
Type 2	Gulkana Glacier	–	2.0	94.0	4.0	–
	Harding Icefield	–	50.0	50.0	–	–
Type 3	Gulkana Glacier	–	–	18.5	81.5	–
Type 4	Harding Icefield	–	–	–	–	100

### SEM

**Figure 5:**
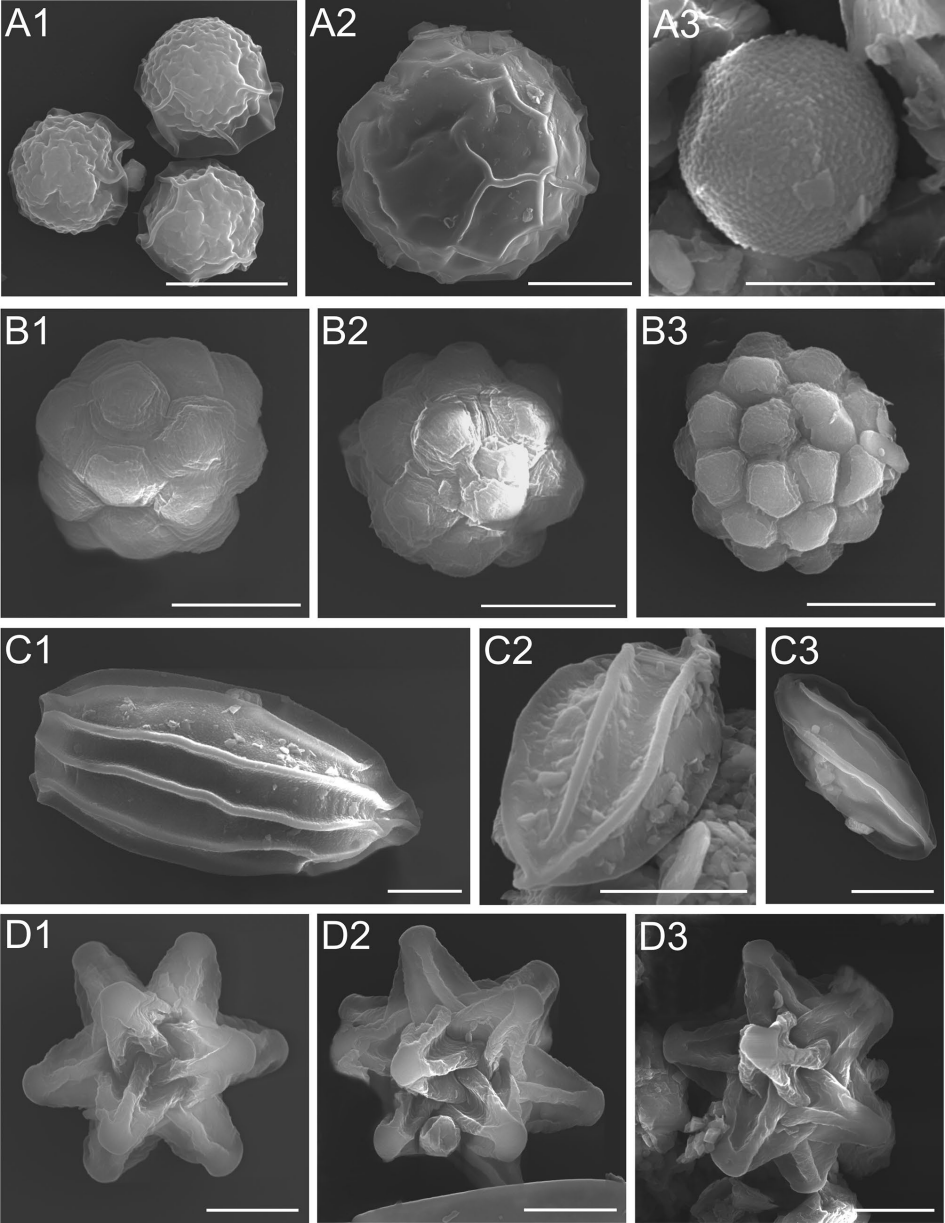
SEM images of different forms of snow algae. (**A1**–**A3**) Round form-spore cells; the outermost layer of the cell wall is wrinkled, (**B1**–**B3**) rosette form-hypnozygote; cell wall flange extending from pole to pole, (**C1**–**C3**) ellipsoid form-zygote, (**D1**–**D3**) star form. The scale bar corresponds to 10 µm.

Figure 5 shows cell types observed in the samples from Alaska. Images marked with A show Type 1, i.e. cyst cells surrounded by a transparent mucous cover. The surface of the cells is rough, and the degree of roughness varies between individual cells (Fig. 5A1,A2). Figure 5A3 shows a cyst with pronounced papillae on the cell surface. Type 2 cells, i.e. hypnozygotes, are characterized by a striking symmetry of their cell wall surface structures. There are numerous sections resembling pentagonal shields, which adhere to each other, thus forming a geometrically ideal form. These surfaces can be flat or more convex, looking like plugs protruding from a regular ball (Fig. 5B1–B3). Type 3 cells are characterized by the presence of longitudinal flanges on the surface, which extend throughout the entire length of the cells from pole to pole and every second flange is connected to the poles (Fig. 5C1–C3). Type 4 is a very striking form built of over a dozen symmetrical arms. Each of the arms is built of five linear scaffoldings. The arms are arranged symmetrically and directed at the same angle in space. They have the same length and the spaces between them are of equal depth (Fig. 5D1–D3).

### Profilometric analysis of algal cells

**Figure 6:**
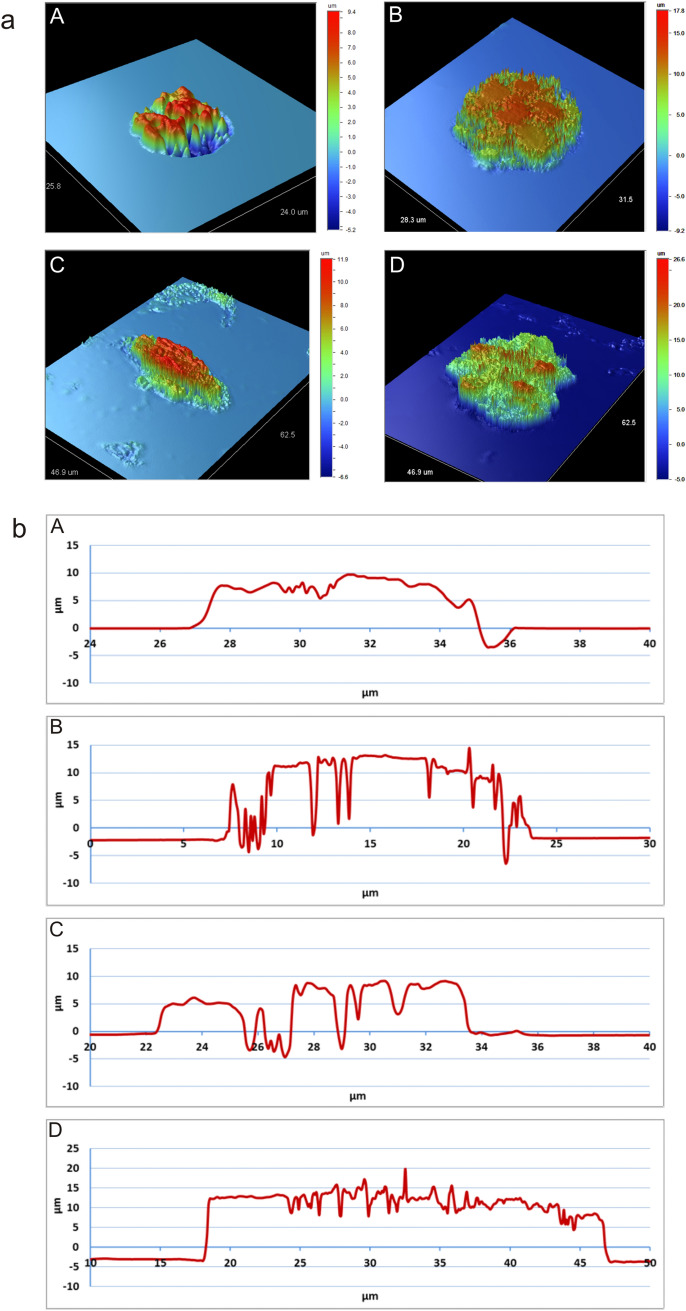
Profilometric analyses of the forms of red snow algal cells. A—round form-spore cell, B—rosette form-hypnozygote, C—ellipsoid form-zygote, D—star form. *R*_*a*_ the arithmetic mean of the elevation profile, *R*_*q*_ the mean square elevation profile, *R*_*t*_ the maximum height of the profile.

The profilometric analysis facilitated the preparation of microgeometry maps of the entire algal surface (Fig. 6a). The tests were carried out for the four forms: Type 1 (Fig. 6a A), Type 2 (Fig. 6a B), Type 3 (Fig. 6a C), and Type 4 (Fig. 6a D). The generated microgeometry maps of the entire algal surfaces allowed determining the following roughness parameters: the arithmetic mean of the elevation profile R_a_, the mean square elevation profile R_q_, and the maximum height of profile R_t_. The study has shown the largest surface roughness in Type 4. The arithmetic mean of the profile ordinates determined for this form is R_a_ = 5.01 µm. The maximum profile height determined for this type of algae also has the highest value, i.e. R_t_ = 31.60 µm. The lowest surface roughness is exhibited by Type 1, which is also confirmed by SEM. The arithmetic mean of the ordinates of the profile determined for this form is R_a_ = 0.76 µm. Similarly, this type of algae also has the lowest maximum profile height, i.e. R_t_ = 14.64 µm. The profilometric analysis also facilitated the determination of the length of the algae tested for the X axis. The following length values were measured: Type 3–12 µm, Type 1–9 µm, Type 4–29 µm, and Type 2–17 µm. The profiles of individual algae in the X axis are shown in Fig. 6b. The roughness parameters of four types of algal cell are presented in Table 4.

**Table 4:** Roughness parameters of four types of algal cells.

Type 1 (µm)	Type 2 (µm)	Type 3 (µm)	Type 4 (µm)
R_a_ = 0.76 ± 0.076	R_a_ = 3.23 ± 0.076	R_a_ = 1.08 ± 0.076	R_a_ = 5.01 ± 0.076
R_q_ = 1.65 ± 0.076	R_q_ = 4.44 ± 0.076	R_q_ = 2.06 ± 0.076	R_q_ = 6.38 ± 0.076
R_t_ = 14.64 ± 0.076	R_t_ = 27.00 ± 0.076	R_t_ = 18.52 ± 0.076	R_t_ = 31.60 ± 0.076

*R*_*a*_ the arithmetic mean of the elevation profile, *R*_*q*_ the mean square elevation profile, *R*_*t*_ the maximum height of profile.

### Cell autofluorescence

**Figure 7:**
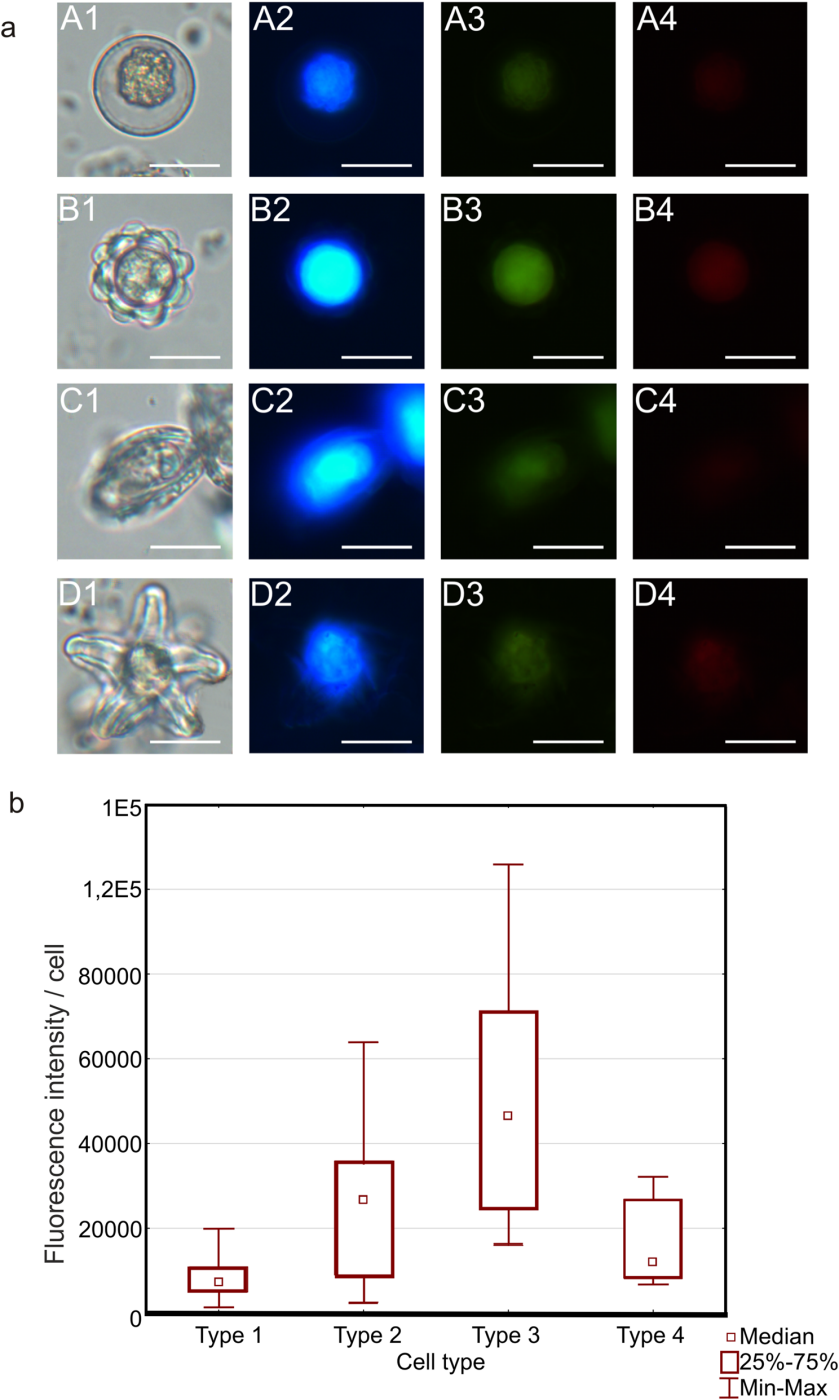
Autofluorescence of various forms of snow algae. Fluorescence observed during exposure to different wavelengths: 365 nm (blue), 470 nm (green), 546 nm (red). (**a**) Images of different forms of algal cells. The scale bar corresponds to 20 µm. (**b**) Analysis of blue fluorescence intensity of different types of algal cells. Kruskal–Wallis, H (3, N = 288) = 111.8398; p = 0.000. Statistically significant differences: Type 1 vs. Type 2 (p < 0.001), Type 1 vs. Type 3 (p < 0.001), and Type 2 vs. Type 3 (p < 0.05).

The autofluorescence of algal cells showed a more intense signal at the 365 nm filter (blue fluorescence), weaker glow at excitation of 470 nm (green fluorescence), and the weakest fluorescence at 546 nm (red fluorescence). The analysis conducted with the use of the three different filters demonstrated the strongest blue fluorescence for all types of algal cells. Types 2 and Type 3 emitted the most intense glow, and Type 1 exhibited weaker fluorescence intensity, similar to Type 4 (Fig. 7a).

Statistical analyzes were performed for fluorescence at excitation at 365 nm for the four cell types. The number of cells that were analyzed was 288 (ANOVA Kruskal–Wallis test for nonparametric analysis. Kruskal–Wallis, H (3, N = 288) = 111.8398; p = 0.000). The median (RFU) was 7248 for Type 1, 26,600 for Type 2, 46,547 for Type 3, and 12,078 for Type 4. Statistically significant differences were found in three cases: Type 1 vs. Type 2 (p < 0.001), Type 1 vs. Type 3 (p < 0.001), and Type 2 vs. Type 3 (p < 0.05). Statistical analysis of the fluorescence values for the individual forms of algae is shown in Fig. 7b.

### Application of fluorochromes

**Figure 8:**
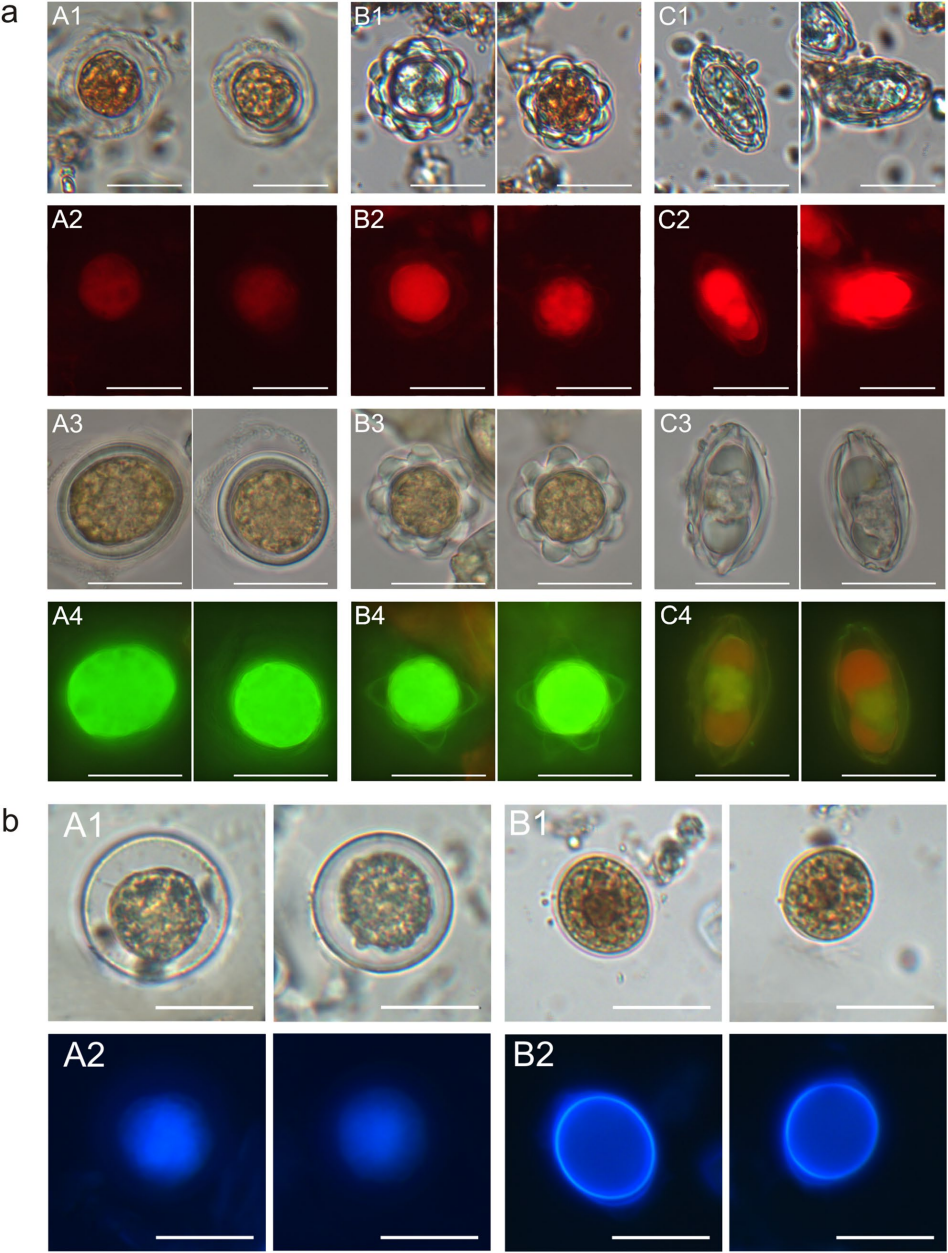
Fluorescence of the different forms of red snow algae after application of fluorochromes. (**a**) Congo red and Acridine orange: the varied intensity of the red fluorescence of the cell walls indicates differences in the structure of their polysaccharide layer (**A2**–**C2**). The green and red fluorescence of the cells indicate the pH of the cells (**A4**–**C4**); (**b**) Calcofluor white: the blue fluorescence of the cell walls indicates the presence of a thick cellulose-type layer in the cell wall, in contrast to cells that do not have the layer (**A2**,**B2**). The scale bar corresponds to 20 µm.

It was observed that Congo red dye bound to the walls of all four cell types. It was also noted that the intensity of light differed between individual cases. Type 1 emitted the weakest red fluorescence, which was slightly stronger in Type 2, and the strongest red fluorescence was observed in Type 3. This may indicate different amounts of polysaccharides in the cell walls of the snow algae (Fig. 8a A2–C2).

After staining the algal cells with the Acridine orange dye solution, all cell types emitted green fluorescence, while the cells of the *Chloromonas* cysts (Type 3) additionally emitted orange light (Fig. 8a A4–C4). The results indicate that all cells have acidic pH.

Figure 8a A1,A3 show round forms of algal cells differing in the cell wall thickness. It was checked whether the Calcofluor white fluorochrome binds to the cell wall of any of the analyzed forms. After staining the cyst cells with Calcofluor white, it was observed that cells with prominently thickened walls stained in a different way than those with thinner walls. The thick walls emitted very bright blue fluorescence, and a glowing border was visible around the cells with a thick wall. In the case of cells with thinner walls, poor fluorescence was only observed inside the cell, whereas no cell wall fluorescence was observed (Fig. 8b). The observations of thick cell wall fluorescence in the round-type algal cells after Calcofluor white staining is described for the first time.

### FTIR spectroscopy and SEM imaging of polysaccharides

**Figure 9:**
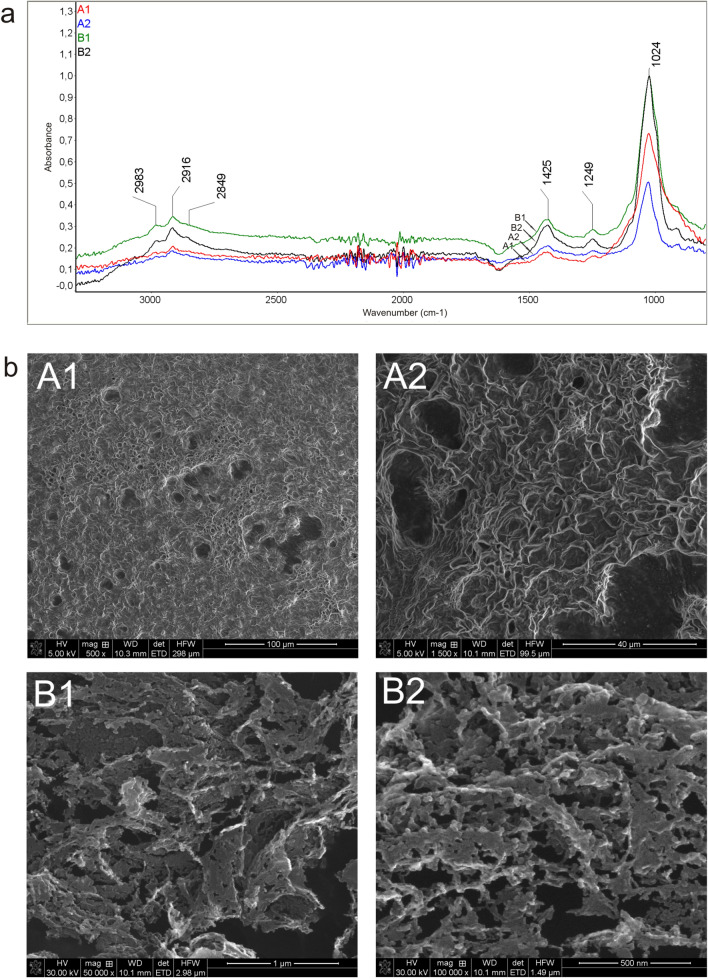
(**a**) FTIR analysis of algal suspension (pellet) (**A1**—sample from Gulkana Glacier, B1—sample from Harding Icefield) and supernatant after separation (**A2**,**B2**). (**b**) SEM analysis of polysaccharide fraction (**A1**,**B2**); polysaccharide chains are marked with arrows.

After staining with Congo red, the dye bound to cell wall polysaccharides and the algal cells exhibited characteristic red fluorescence with varying intensity depending on the type. To study the presence of polysaccharides in the algae in relation to the sampling sites, FTIR spectroscopic tests were performed. The analysis of the algal suspensions from the two sites showed the presence of a characteristic band at a position of 1434 cm^−1^ corresponding to deformation vibrations of C–OH groups and symmetrical stretching vibrations of O–C–O groups derived from carboxyl groups. The FTIR spectra of the samples showed the presence of a low-intensity peak at 1246 cm^−1^ corresponding to C–O deformation vibrations and an intense 1024 cm^−1^ peak corresponding to C–C stretching vibrations, probably derived from pyranose rings, which may suggest the presence of oligosaccharides. The FTIR analyses also showed the presence of C-H stretching vibrations in the CH_3_ and CH_2_ groups in the wavenumber range 2852–3009 cm^−1^. These bonds may indicate the presence of lipids in the tested algae. The Harding Icefield algae were characterized by a substantially higher intensity of the peaks in the range of 2852–3009 cm^−1^ (Fig. 9a). The algal suspension supernatant was also analyzed and the solvent spectrum was subtracted. Similar spectra were observed as in the analysis of the algal cells, with peaks at the same position (Fig. 9a). A more intense peak was observed for algal cells from Harding Icefield than those from Gulkana Glacier. These observations indicate that the polysaccharides penetrated into the solution. The SEM images of the freeze-dried preparations from Harding Icefield showed characteristic polysaccharide structures (Fig. 9b). Especially at high magnifications of 50,000 × and 100,000 ×, polysaccharide chains with granules connected in series were observed (marked with arrows) (Fig. 9bB1,B2).

### SEM/energy dispersive X-ray spectroscopy (EDS) analysis of polysaccharides

The elemental composition of the lyophilized samples was analyzed and compared with each other (Table 5). Four measurements were carried out for each sample. The elemental composition in all analyzed samples supported the hypothesis that they mainly contain polysaccharides. The dominant elements of the analyzed samples were carbon and oxygen or sugar-building elements. The carbon and oxygen content was 43.9% and 51.6%, respectively, in the sample from Gulkana Glacier and 48.9% and 50.5%, respectively, in the Harding Icefield sample (Table 1). The content microelements of such as Na, Mg, Al, Si, S, Cl, K, Ca was in all cases below 1%, but was definitely higher in the sample from the former glacier. Nitrogen at 1–2% was found in both samples but not at each site analyzed. Iron was found only in the first sample (Table 5).

**Table 5:** Elemental composition of the polysaccharide fraction of snow algae in five samples (S1–S5): S1—Gulkana Glacier, S2—Harding Icefield.

Element	Gulkana Glacier						Harding Icefield					
	# 1	#2	#3	#4	Average	SD	# 1	#2	#3	#4	Average	SD
C	45.97	43.78	43.73	41.97	43.86	± 1.42	49.92	49.07	47.95	48.81	48.94	± 0.70
N	0.00	0.00	2.02	2.13	1.04	± 1.04	0.00	0.00	1.01	0.00	0.25	± 0.44
O	50.96	52.34	51.12	52.00	51.61	± 0.58	49.74	50.62	50.9	50.9	50.54	± 0.48
Na	0.36	0.45	0.38	0.45	0.41	± 0.04	0.08	0.07	0.05	0.05	0.06	± 0.01
Mg	0.30	0.35	0.34	0.35	0.34	± 0.02	0.03	0.03	0.03	0.05	0.04	± 0.01
Al	0.23	0.27	0.27	0.25	0.26	± 0.02	0.08	0.07	0.11	0.03	0.07	± 0.03
Si	0.47	0.55	0.58	0.54	0.54	± 0.04	0.10	0.07	0.07	0.11	0.09	± 0.02
S	0.59	0.82	0.50	0.86	0.69	± 0.15	0.00	0.01	0.01	0.07	0.02	± 0.03
Cl	0.05	0.06	0.04	0.06	0.05	± 0.01	0.01	0.01	0.01	0.01	0.01	± 0.00
K	0.14	0.16	0.15	0.18	0.16	± 0.01	0.02	0.02	0.02	0.02	0.02	± 0.00
Ca	0.81	1.06	0.74	1.09	0.93	± 0.15	0.01	0.02	0.02	0.01	0.02	± 0.01
Fe	0.12	0.15	0.13	0.12	0.13	± 0.01	0.00	0.00	0.00	0.00	0.00	± 0.00

### X-ray photoelectron spectroscopy (XPS)

**Figure 10:**
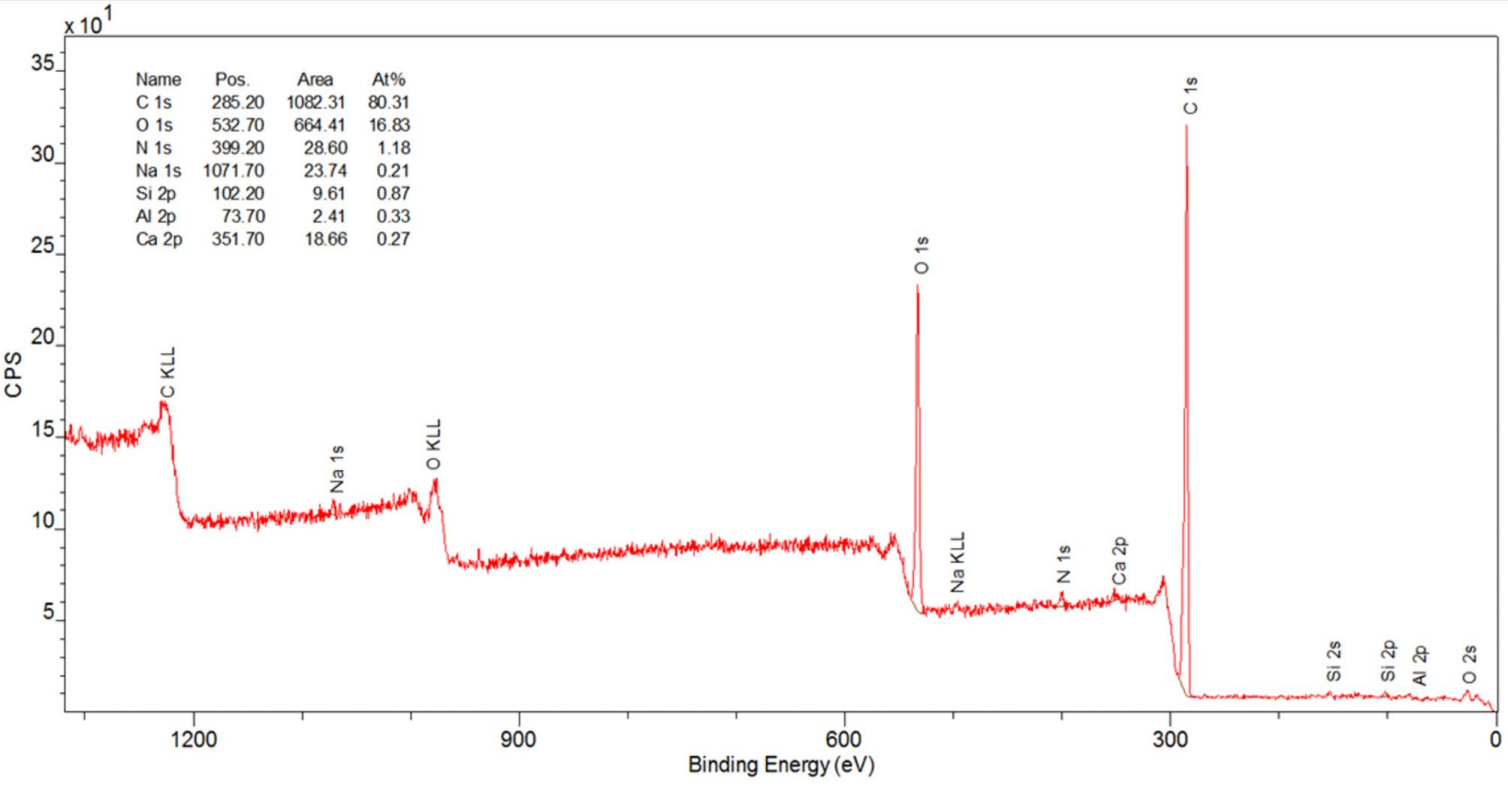
XPS spectrum of the polysaccharide fraction of the snow algae from the Gulkana Glacier.

The XPS analyses carried out in a wide range of binding energy showed the presence of the following elements: carbon, oxygen, nitrogen, sodium, silicon, aluminum, and calcium. The XPS spectrum is shown in Fig. 10. Analyses were also performed in a narrow range of binding energy characteristic of carbon. These data of the sample from the Gulkana Glacier demonstrated 25.4% of carbon in the form of aliphatic C–C bonds, 24.5% of carbon in the form of aliphatic C–H bonds, 18% of carbon in the form of aromatic C=C bonds, and 12.8% of carbons in the form of C–OH bonds. These bonds are characteristics of algal polysaccharides and oligosaccharides. Based on the tests, the presence of such groups as C–O–C and O=C–O– was found. The presence of these groups was also confirmed by FTIR spectroscopy. Table 6 contains the results of the XPS analyses.

**Table 6:** Results of XPS analysis of the polysaccharides fraction in the snow algae from the Gulkana Glacier.

Name	Position	Raw area	% at conc.	Species	Phase
C 1s A	284.48	38,832	18.8	C=C	Aromatic carbon
C 1s B	285.01	50,705	24.5	C–H	Aliphatic carbon
C 1s D	285.58	52,504	25.4	C–C	Aliphatic carbon
C 1s E	286.42	26,535	12.8	C–OH	Hydroxyl groups
C 1s F	287.14	11,910	5.8	C–O–C	Ether groups
C 1s G	287.86	4831	2.3	C=O	Carbonyl groups
C 1s H	288.99	19,468	9.4	O=C–O–	Carboxyl groups
C 1s I	290.17	2109	1.0	CO_3_^–2^	Carbonates

## Discussion

Since snow algae are adapted to living in an environment with low temperatures in the alpine or polar regions, they possibly produce a number of compounds such as sugars, sugar alcohols, and lipids and form mucous casings and cysts to adapt to such environmental conditions. Both the features of the structure and the compounds produced by the algae contribute to their survival and development as well as spread over large areas.

### Suggested identification of taxa based on cell morphology

Three different types of algal cell were found in the snow fields of each glacier. DNA sequencing of 18S rRNA gene and ITS2 regions of red-snow samples showed that Chlamydomonas-snow-group-B (conspecific with *Chlamydomonas nivalis* or *S. nivaloides*) accounts for 95% of the total sequences for Gulkana Glacier, while Chloromonadinia-snow group G (conspecific with *Chloromonas* sp.) and Chlamydomonas-snow-group-B account for 75% and 20%, respectively for Harding Icefield^34^. Based on the sequence data, Types 1 and 2 cells are likely to be *S. nivaloides* and Type 4 is likely to be *Chloromonas* sp. (Chloromonadia snow group G), which was identified by single cell sequencing^34^. Type 3 appears to be *Chloromonas* sp. (Chloromonadia snow group A, which accounts for 4% of the total sequences of Gulkana Glacier^34^). The morphological observations allow the putative determination of *S. nivaloides*. In the obtained image, *S. nivaloides* was identified based on the characteristic papules on the cell wall surface. The structure of these cells has been described and illustrated by Procházková et al.^15^. Figure 5A3 showed cyst with pronounced papillae on the cell surface, which is a characteristic of *S. nivaloides*^15^. The microscopic observations revealed that as much as 58% of the Harding Icefield cysts are represented by *S. nivaloides*. The scanning electron microscope made it possible to observe the regular rosettes of Type 2. Type 2 cells are similar to snow algal cells of hypnozygotes observed by Müller et al.^6^ with the use of SEM. Type 3 resembles the algal cells of *Chloromonas nivalis* shown in the publication by Muramoto et al.^32^ or Remias et al.^33^, whose zygote was characterized by an identical arrangement of collars, extending to two poles. Despite the striking morphology, Type 4 was not identified to the species level. Type 4 cells are the largest algae found in the analyzed samples. Data on the star form identified as *Chloromonas* spp. are limited; so far, this form has been visualized and described by Segawa et al.^34^. All four types of algae cells and their taxonomic assignment are presented in Table 2. In order to determine accurately the species affiliation of each of the presented forms of algae, DNA analysis should be performed using high technique cell manipulation for each cell. Such studies are planned in the future.

### Polysaccharides in the algal cell wall

The four types of cells also had a diverse composition of complex polysaccharides, which is likely crucial for their function and survival. Polysaccharides contained in the cell wall are secreted outside to protect the cells from severe frost. Congo red is mostly used to dye glucans, which comprise a group of β-D-glucose polysaccharides naturally occurring in the cell walls of bacteria^35,36^ and fungi^24,37^. Our observations of cells stained with Calcofluor white dye suggest that there is cellulose-type compound in the algal cell wall that react with the dye and give off intense blue fluorescence.

Congo red binds to the outer layer of the algal cell wall containing sulfated polysaccharides. The observations of the different intensity of the red fluorescence of cell walls depending on the developmental form of the red algae analyzed revealed differences in the wall structure. Congo red forms a complex only with a polymer, and the spectral changes associated with the intensity of red glow are dependent on the properties of the polysaccharides that form polymers in the cell wall^36^. Type 3 of algal cells shone most intensively, which indicates that the polymers forming the dye complex are located in the outer layer of the cell wall.

Calcofluor white is a fluorescent blue dye used to bind to the polysaccharide polymers of the cell walls in different organisms. It functions by binding to β-(1-3) and β-(1-4) polysaccharides on chitin and cellulose present in the cell walls on fungi, plants, and algae^29,38^. Calcofluor white was also used to stain Type 1 *Sanguina* sp. cells. *S. nivaloides* can be distinguished from other red algal cysts by the number of cell wall layers, cell size, cell surface morphology, and habitat preferences. This alga has a thicker three-layer cell wall, in contrast to *S. aurantia*, whose cell wall is composed of two layers and contains fine granules^15^. Similar cells with a thick cell wall classified as *Chloromonas nivalis* were described by Remias et al.^39^ and Holzinger et al.^40^. Since the dye binds to the cell wall of one of the analyzed species, forming a bright glowing envelope of the thick cell wall, this staining method can be helpful in distinguishing species of red snow algae. Calcofluor white was used to stain the cell walls of marine green algae by other researchers^41^. Staining with this fluorochrome showed accumulation of cellulose on the surface of the primary envelope. We obtained a similar image of the red algal thick-walled cells after staining, which may suggest that the cellulose layer of the cell wall emits fluorescence in cells with a thicker cell wall. After staining other types of algae using Calcofluor white, no blue glowing structures were observed, but there was only fluorescence enhancement causing green-yellow glow of the cells.

The FTIR spectroscopy studies showed the presence of polysaccharides not only in the algal cells but also in the solution in which the cells were fixed, which indicates the release of these compounds outside the cells. Extracellular polymeric substances may also constitute the algal extracellular matrix^42^. We observed peaks in the same positions for both the cell suspension and the solution in which they were located. There were peaks in the same positions but with different intensities. The high intensity of the peak of the sample from site 2—Harding Icefield corresponding to the binding present in polysaccharides may be associated with the presence of the star-shaped algae, which were not observed in the sample from Gulkana Glacier. It is worth adding that, although this form was not present in large quantities, this cell is larger than the other algal cells and may thus substantially influence the spectrum intensity. However, this is a rare form, which makes it difficult to analyze. The X-ray photoelectron spectroscopy (XPS) analyses confirmed the presence of polysaccharides in the tested sample. Our studies have shown the presence of polysaccharides released from the cells in the formalin solution in which the cells were fixed. This is a valuable observation, as the compounds can be obtained in this way for treatment of human pathogens or other medical applications^43^. The research on polysaccharides contained in red snow algae will be continued.

### Ecological aspect

To date, there are no data on the comparison of autofluorescence of individual forms of algal cells. The cells emit fluorescence upon excitation with certain wavelength light. On Gulkana Glacier, specific absorption was observed in the spectrum at wavelength ranges of 400–600 and 670–680 nm, corresponding to the red color of the snow surface caused by snow algal blooming^44^. The observations presented by Takeuchi were confirmed by Holzinger et al.^40^, who reported the highest absorption in the cells of *Chlamydomonas nivalis* in the wave band between 400 and 600 nm and an additional peak at around 680 nm. In our studies, the algal cells were observed with the use of optical filters with a wavelength of 365 nm, 470 nm, and 546 nm. These wavelengths are close to the range mentioned above. Algae in red snow have photosynthetic chlorophyll, which is green, and contain astaxanthin, i.e. a secondary red carotenoid pigment protecting algae from ultraviolet light. Chlorophylls show two distinct absorption maxima: one between 400 and 500 nm and the other between 600 and 700 nm. The absorption maxima of carotenoids can expand over 500 nm. It has been revealed that secondary carotenoids show absorbance between 400 and 550 nm^40^. Excited chlorophyll dissipates absorbed light energy by driving photosynthesis as heat in a protective mechanism against excessive radiation (photochemical quenching) or by emission of fluorescent radiation. Chlorophyll fluorescence analysis is an important research tool with the potential of application^45–48^. In our observations, the zygote forms (hypnozygote) showed the strongest blue fluorescence while absorbing light at the wavelength of 365 nm. The light intensity caused by blue emission of the zygotes is clearly higher than that of the other cells. Investigations of the observed effect will continue.

The microelements contained in the polysaccharide fraction can be derived from organic particles, accompanying microorganisms secreting their metabolites outside, and algal cells themselves. The cell wall of red snow algae is a rigid shield, difficult to destroy mechanically even in extreme low temperature conditions. The mucus covering the cell walls facilitates adhesion of inorganic and organic substances and microorganisms^6^. Such structures as fungi, bacteria, or dust particles are often attached to the outer wall of algal cells^19^. The main elements of particles attached to the cell wall are Si, Al, Fe, and O, regardless of the origin of the snow algae, and the same elements have been found in cell vacuoles^49^. This suggests that the uptake of the relevant elements takes place through the mechanism of pinocytosis. The combined transport of toxic aluminum with silicon may be unavoidable, because the consumption of inorganic nutrients from snow algae is limited to a thin layer of water between snow grains. However, the formation of insoluble aluminum silicates can be considered a detoxification mechanism^49^. The content of elements, mainly nitrogen^4,50^ but also P, S, K, and Fe, affects the pigmentation of snow algal cells^6,50^. Snow usually contains very small amounts of nutrients, mainly introduced by air, dust, and precipitation. The inorganic particles present on the surface of snow algal cells may be important for their survival^49,51^.

In conclusion, the red snow algal cells found from two locations in Alaska during the melting season differ in morphological and physiological terms. The cells emitted varied fluorescence depending on the form and size of the cell, which can be used in environmental research. The different forms of the algae differ in the structure of the polysaccharide layer in the wall. The analysis revealed the presence of extracellular polysaccharides from algal cells. Cell fluorescence induced using fluorochromes may be helpful in identifying species with different cell wall structures, and the method for obtaining exopolysaccharides may be used in the future to analyze their usefulness in medicine.
